# Implication of Oxysterols in Infectious and Non-Communicable Inflammatory Diseases

**DOI:** 10.3390/cells12020241

**Published:** 2023-01-06

**Authors:** Gérard Lizard, John J. Mackrill, Tim Willinger

**Affiliations:** 1 Team “Biochemistry of the Peroxisome, Inflammation and Lipid Metabolism”, Université de Bourgogne Franche-Comté/Inserm, 21000 Dijon, France; 2 Department of Physiology, School of Medicine, University College Cork, Western Gateway Building, Western Road, T12 XF62 Cork, Ireland; 3 Center for Infectious Medicine, Department of Medicine Huddinge, Karolinska Institutet, 141 52 Stockholm, Sweden

Oxysterols, derived from cholesterol oxidation, are formed either by autoxidation, via enzymes, or by both processes [1,2]. These molecules have multiple biological activities and can regulate oxidative stress, inflammation, cell death, as well as cell differentiation and cholesterol homeostasis [3,4,5]. At the cellular level, depending on their structures, oxysterols can act at the level of the plasma membrane, endoplasmic reticulum, organelles (mitochondria, peroxisome and lysosome) and/or at the nuclear level. Several of these oxysterols, in particular those resulting from the oxidation of cholesterol on its side chain, can be ligands or activators of the following receptors: (i) nuclear receptors, such as liver X receptors (LXRs) α or β [6] and retinoic acid receptor-related orphan receptor α and γ (RORα [NR1F1] and RORγ [NR1F3]) [7], but also (ii) cytoplasmic receptors such as SREBP (sterol regulatory element binding transcription protein) [8], NPC1 (NPC intracellular cholesterol transporter 1 / Nieman-Pick type C1) [9], FXR (NR1H4, farnesoid X receptor alpha) [10], oxysterols binding proteins (OSBPs), OSBPs-related proteins (ORPs) [11,12] and cholesterol epoxide hydrolase (ChEH) (also named anti-estrogen binding site (AEBS); ChEH is an hetero-oligomeric complex comprising 3beta-hydroxysterol-delta(8)-delta(7)-isomerase (D8D7I) and 3beta-hydroxysterol-delta (7)-reductase (DHCR7)) [13] as well as (iii) membrane receptors such as receptor tyrosine kinases [14] and the Epstein–Barr virus-induced gene 2 receptor (EBI2, also known as GPR183) [15,16,17]. Some of these receptors are involved in the control of cholesterol trafficking, cell proliferation, and cell death. For the receptors (RORs, FXR, LXRs, EBI2), there are several lines of evidence for their involvement in inflammation [18,19,20,21]. Other oxysterols oxidized at C7, such as 7-ketocholesterol (7KC) and 7β-hydroxycholesterol, which either minimally or do not interact with these receptors, are potent inducers of inflammation and are known to have an important role in the pathophysiology of many age-related diseases (cardiovascular, ocular and neurodegenerative diseases) [3,22]. These C7-oxidized oxysterols trigger both the production of inflammatory cytokines [23] and prostaglandins [24,25]. Prostacyclin (PGI2) production, which promotes platelet aggregation, has also been described in 7KC-treated endothelial cells [26]. The ability of 7KC to induce inflammation is likely to occur mainly through the TLR4 receptor both in vitro and in vivo [27]. 

To date, the pro-inflammatory activities of oxysterols are thought to be involved in chronic inflammatory diseases (cardiovascular diseases, inflammatory bowel disease), as well as in common (multiple sclerosis, Alzheimer’s disease) and rare neurodegenerative diseases, such as X-linked adrenoleukodystrophy (X-ALD) [22,28,29,30]. Certain oxysterols can also act on bacteria, viruses, and parasites [31,32,33]. Thus, several oxysterols are involved in the immune response and can act on infectious agents [34]; their involvement in the immune response and cytokine storm is very likely, because some of their receptors are associated with immune activities and signaling pathways by which oxysterols promote cytokine production. The aim of this Special Issue is to cover these different aspects as well as pharmacological studies on the molecules that modulate the biological activities of oxysterols in both infectious and non-communicable inflammatory diseases. This Special Issue entitled “Oxysterols and the Immune Response: Implications in Non-communicable and Infectious Diseases” was supervised by three Guest Editors: Dr John Mackrill (University College of Cork, Cork, Ireland), Dr Tim Willinger (Karolinska Institutet, Stockholm, Sweden) and Dr Gérard Lizard (University of Burgundy/Inserm, Dijon, France). Five publications are associated with this Special Issue including three reviews and two research papers.

The review by Fabio Alessandro de Freitas et al. [35] focuses in particular on 25-hydroxycholesterol and 7α,25-dihydroxycholesterol in the immune system and related diseases. The effects of these oxysterols and the LXRs and EBI2 receptors are discussed in the context of the immune response in the blood and central nervous system. The implication of these oxysterols in several chronic inflammatory diseases and certain cancers are also presented. The review by Cheng Xiang Foo et al. [36] covers the state of knowledge regarding oxysterols and their effect on the control of intracellular bacterial growth as well as viral entry into the host cells and viral replication. The review by Lisa Reinmuth et al. [37] surveys the two broad classes of cell-surface receptors for oxysterols (G protein-coupled receptors and ion channels), the mechanisms by which oxysterols act on them, and their functions in the different cell types of the immune system. In addition, Line Barington et al. [38] showed that GPR183/EBI2 is unnecessary for B1 cell accumulation and function, but affects B2 cell abundance, in the omentum (fatty tissue, part of the peritoneum, connecting stomach, intestine and colon) and peritoneal cavity. Furthermore, when human brain endothelial cells (hCMEC/D3) were cultured in the presence of 7KC, an increase in the expression of pro-inflammatory cytokines (IL-1β, IL-6, IL-8 and TNF-α) was observed as well as an increase in the expression of cyclo-oxygenase-2 (COX-2) which catalyzes the conversion of arachidonic acid to prostaglandins [39]. In the presence of withanolide A, a naturally occurring phytochemical that is found in Ashwagandha (Withania somnifera, fam. Solanaceae) and Indian Ginseng, the pro-inflammatory effects of 7KC were strongly and significantly reduced as well as its pro-oxidative effects associated with cell death induction [40]. 

This Special Issue of Cells, therefore, presents several works that provide information to better understand the pro-inflammatory effects of several oxysterols and some of their associated receptors, both in the context of infectious diseases and chronic inflammatory diseases with potential pharmacological applications.
